# The Implementation Gap in Early Septic Shock Resuscitation: A Three-Barrier Framework

**DOI:** 10.3390/jcm15124572

**Published:** 2026-06-12

**Authors:** Sajid Kadir, Travis Murphy, Joseph Shiber

**Affiliations:** 1 Divisions of Cardiovascular Medicine and Pulmonary and Critical Care Medicine, University of Florida College of Medicine—Jacksonville, Jacksonville, FL 32209, USA; 2 Department of Surgery, University of Miami, Miami, FL 33136, USA; 3 Department of Emergency Medicine, University of Florida College of Medicine—Jacksonville, Jacksonville, FL 32209, USA

**Keywords:** septic shock, norepinephrine, vasopressors, implementation, emergency department, SEP-1, quality measures

## Abstract

The case for early vasopressor initiation in septic shock has been argued in detail in physiologic reviews and randomized trials. The evidence base is no longer the limiting factor. What remains limiting is delivery. Across most U.S. emergency departments and many international settings, patients with septic shock still do not reliably receive norepinephrine within the first hour of recognition. This review reframes the early-vasopressor question from a physiologic argument into an implementation problem and identifies three structural barriers that operate independently of any individual clinician’s understanding of the underlying evidence. The first is regulatory: the SEP-1 quality measure, despite a documented physician exception for the fluid requirement, continues to incentivize a fluids-first sequence as the institutional default. The second is cultural: the gap between policies that permit peripheral norepinephrine administration and the workflows, scope-of-practice arrangements, and standing orders required to actually start it at the bedside. The third is upstream: time-to-vasopressor is partly a downstream surrogate for time-to-recognition, and interventions that target only the pressor decision miss the larger source of delay. We propose a parallel resuscitation framework with explicit protocolized triggers and stratify implementation considerations across U.S. academic centers, U.S. community emergency departments, and resource-limited international settings. Closing the gap means stopping the physiology argument and rebuilding the operational architecture.

## 1. Take-Home Message

The contemporary obstacle to early vasopressor therapy in septic shock is not what clinicians believe about the evidence but the regulatory, cultural, and recognition-stage structures within which they work. Addressing those structures, rather than re-arguing the physiology, is the field’s most consequential remaining task.

## 2. Introduction

Sepsis and septic shock remain among the leading causes of death worldwide, and case fatality in septic shock has fallen only modestly over the past decade despite sustained research investment [1,2]. The argument for early vasopressor initiation in septic shock has been made repeatedly across more than a decade of literature. This evidence base is heterogeneous. Only some studies, such as the CENSER trial [3] and propensity-matched cohorts [4], tested vasopressor timing directly; others, including CLOVERS [5], CLASSIC [6], and PLUS [7], addressed fluid strategy or composition rather than norepinephrine timing. Taken together with a recent emergency department cohort from Bogotá [8], they point in a consistent direction rather than providing uniform, timing-specific proof. In septic shock, delayed restoration of vascular tone is associated with worse outcomes, and the historical sequence of mandatory crystalloid loading before vasopressor consideration is not supported by current evidence. This article uses physiologic and trial-evidence case as its premise and integrates a focused review of the vasopressor-timing evidence with an implementation-science lens; it is intended as conceptual and implementation-oriented commentary rather than a systematic review. Throughout, we use ‘early’ to mean vasopressor initiation within approximately the first hour of septic shock recognition and concurrent with, rather than after, initial fluid resuscitation. We define recognition as the first documented clinical identification of septic shock, meeting operational criteria of infection with hypotension, hypoperfusion, or lactate ≥ 4 mmol/L. The ambiguity of this time-zero, whether anchored to triage, the lactate result, sepsis-alert activation, or physician documentation, is itself part of the recognition problem examined in Barrier 3.

Despite this accumulated evidence, U.S. emergency departments still administer vasopressors with substantial delays, frequently exceeding the first hour after septic shock recognition and in some cohorts extending to several hours [9,10]. The evidence is widely disseminated through major trials and professional guidelines, but practice has not changed in proportion to it. The persistence of this gap, across institutions, regions, and resource levels, suggests that the obstacle is not principally cognitive or evidentiary. It is structural.

This review identifies three structural barriers that delay vasopressor initiation independently of any individual clinician’s beliefs about the underlying physiology, regulatory and quality-measure incentives that codify the fluids-first sequence, cultural and operational uncertainty around peripheral norepinephrine, and the upstream recognition problem for which pressor timing is a partial surrogate. We then propose a parallel resuscitation framework with protocolized triggers and stratify implementation considerations across three care environments. The argument is not that more evidence is needed, but that existing evidence will not change practice until the operational architecture is changed. The framework proposed here is conceptual rather than empirically derived or formally validated; it organizes the existing evidence to locate where delivery, rather than knowledge, fails.

The analysis that follows is centered on adult patients in emergency and critical care settings, and the regulatory, cultural, and recognition-stage barriers are described as they operate in adult practice. Many of the same principles extend to children and neonates, where interest in early vasoactive therapy is also growing. The 2026 Surviving Sepsis Campaign pediatric guidelines, for example, suggest initiating vasoactive medications through peripheral venous access rather than delaying therapy until central access is obtained, mirroring the adult position [11]. The structural barriers, however, are not identical. Pediatric and neonatal septic shock differ in hemodynamic phenotype and they are governed by separate guidelines, weight-based dosing, and quality measures rather than by SEP-1. The vasoactive landscape also differs. Adult guidelines favor norepinephrine as the first-line agent, whereas pediatric guidelines treat epinephrine and norepinephrine as co-equal first-line options and did not favor one over the other [12]. Recognition is harder as well, since hypotension is a late finding in children, and shock must often be identified on clinical grounds before blood pressure falls. The implementation barriers described here should therefore be read as adult-specific in their details, with the pediatric and neonatal versions of the same problems warranting dedicated analysis rather than direct extrapolation [11,12].

### 2.1. The Hemodynamic Premise, in Brief

For completeness, septic shock is dominated by vasoplegia, endothelial dysfunction, and microcirculatory failure, with relative rather than absolute volume depletion in many patients [13,14]. Early α-adrenergic vasoconstriction restores systemic vascular resistance and augments venous return without requiring large-volume crystalloid administration [15]. The physiologic and trial rationale for acting on this earlier rather than later has been argued in detail in the resuscitation literature.

### 2.2. Three Structural Barriers

#### 2.2.1. Barrier 1: A Quality Measure That Penalizes Evidence-Concordant Care

SEP-1 was designed to reduce sepsis mortality. Compliance with the bundle has been associated with lower mortality in large administrative analyses. That association weakens, and in some analyses disappears, once the greater clinical complexity of noncompliant patients is taken into account [16,17]. In the specific domain of early vasopressor therapy, its incentive structure may work against contemporary evidence. The bundle requires 30 mL/kg of crystalloid within three hours and scores compliance as all-or-nothing—fail one element, fail the measure [18]. A physician exception for the fluid requirement allows a lesser volume when the reason is specifically documented [18]. Invoking it reliably, however, requires documentation precise enough to survive abstraction, and because the measure is scored all-or-nothing, any shortfall fails the entire bundle [18]. The practical effect is an institutional incentive to deliver the full 30 mL/kg rather than to rely on case-by-case exceptions, even when a lesser volume is clinically appropriate.

The result is a measurement architecture that may disadvantage physiologically appropriate care. A clinician who limits crystalloid in a patient with obvious vasoplegia and starts norepinephrine at minute ten has done what the evidence supports; that clinician’s institution can still absorb a compliance failure. A clinician who administers the full 30 mL/kg, regardless of hemodynamic phenotype, absorbs none. Over many cases, institutional behavior tends to follow the metric. Quality leaders may hesitate to rely on a fluid exception that protects compliance only when documentation is precise and the chart withstands review.

This creates a tension that the field has been slow to articulate. One of the most widely enforced sepsis quality measures in the United States may, in this specific respect, run counter to the resuscitation strategy that contemporary evidence increasingly supports. The 2026 Surviving Sepsis Campaign guidelines move in this direction, endorsing peripheral vasopressor initiation to avoid central-access delays, allowing early concurrent vasopressor use in unstable patients, and permitting a restrictive fluid strategy after initial resuscitation when continued liberal fluids would otherwise be required [19]. Even so, a guideline that still defaults to 30 mL/kg up front carries limited operational force when the scored metric points in the same fluids-first direction. The authority of the guidelines themselves is, moreover, not uncontested in emergency medicine. In February 2026 the American College of Emergency Physicians declined to endorse the updated Surviving Sepsis Campaign guidelines, citing concern that they did not fully reflect a reality-based approach to emergency department care [20]. Whatever the merits of that position, it underscores that guideline publication does not by itself produce the professional consensus needed to change bedside behavior. Until SEP-1 either incorporates early vasopressor initiation as a valid perfusion-restoration pathway or moves away from all-or-nothing scoring, the measure is likely to remain a significant structural obstacle to practice change in U.S. emergency departments, in part because it operates at the level of institutions rather than individual clinicians.

How institutions and regions respond to this measurement environment varies considerably across the United States. Several states have addressed sepsis through mandated protocols that operate alongside the federal SEP-1 measure. New York was first, with the 2013 regulations known as Rory’s Regulations, which require all acute-care hospitals to adopt sepsis protocols, train staff, and report adherence and outcomes to the state. In adults, this mandate was associated with improved bundle compliance and a reduction in sepsis mortality that outpaced concurrent declines in comparison states, suggesting a real, if modest, effect on outcomes [21,22]. The pediatric experience was different. A parallel analysis across New York and four control states found no significant effect of the regulations on overall pediatric sepsis mortality, with benefit limited to certain subgroups [23]. New York’s approach was followed by similar policies in Illinois and New Jersey, with additional states subsequently enacting legislation [24]. The New York experience is also a useful cautionary tale for the present argument. Its protocols emphasized early fluids and antibiotics, not early vasopressors, and hospitals had to reconcile New York’s reporting requirements with the differently structured federal SEP-1 measure [25].

The broader lesson is that mandates accelerate whatever sequence they encode, and that their measurable benefit is neither uniform across populations nor guaranteed. A regulation that codifies fluids-first will entrench it just as effectively as SEP-1 does. Analyses of why the New York program succeeded also emphasize early engagement of hospitals, physicians, and advocacy groups rather than a top-down design. This suggests that any future measure incorporating early vasopressor initiation would need comparable stakeholder buy-in to change behavior. At the institutional level, responses appear to range from academic centers that build documentation templates to invoke the SEP-1 fluid exception routinely, to community hospitals that default to full crystalloid loading to protect their compliance scores. This is the same divergence in incentive response that the framework below addresses by stratifying its recommendations across care settings.

#### 2.2.2. Barrier 2: The Peripheral Norepinephrine Policy-Practice Gap

The second barrier is the cultural and operational distance between policy and practice on peripheral norepinephrine. Evidence supporting short-term peripheral administration through a proximal large-bore intravenous catheter is now substantial, with low rates of clinically significant extravasation when standard precautions are followed [26]. Major professional society statements, including the 2026 Surviving Sepsis Campaign guidelines, explicitly endorse the practice [19]. Most U.S. academic emergency departments have written policies permitting it. By the standards of guideline-policy concordance, this looks like a solved problem.

The bedside reality is different. Peripheral norepinephrine is permitted almost everywhere. The question is who starts it, on what trigger, through which workflow, and with what pharmacy support. Central venous access has traditionally been treated as a prerequisite for vasopressor administration [27], and that expectation can persist even when peripheral access is already in place, deferring norepinephrine until a central catheter is obtained [28]. Pharmacy dispensing constraints, nursing scope-of-practice variation across shifts, and the absence of standing orders for peripheral pressor initiation all introduce friction that compounds into clinically meaningful delays. The composite result is a system in which peripheral norepinephrine is theoretically endorsed and operationally unavailable.

Closing this gap takes four unglamorous, institutional changes. Standing orders should specify the mean arterial pressure (MAP) threshold that triggers peripheral norepinephrine. Nursing protocols should route the order to pharmacy and bedside infusion preparation in parallel, not in sequence. In-service education should frame peripheral pressor initiation as routine rather than exceptional. And pharmacy stocking should allow an infusion to be prepared within minutes of order entry. None of these interventions are conceptually novel.

The same protocols should make the safety parameters explicit, since operational confidence depends on a clear, shared standard of practice rather than case-by-case judgment. Published peripheral-vasopressor protocols converge on a recognizable set of precautions. Specifying them reduces both risk and hesitation. Catheters should be of adequate caliber, typically 18 to 20 gauge, and sited in a proximal large vein of the forearm or upper arm, avoiding the hand, wrist, and other distal locations where extravasation and tissue injury are more likely [26,29]. The infusion site should be assessed at defined intervals, commonly every one to two hours during active infusion [29,30]. Many protocols cap the dose and duration of peripheral administration, commonly limiting it to roughly 24 to 48 h, and transition to a central line when higher or more prolonged support is anticipated [29]. An extravasation response should be pre-specified rather than improvised—stop the infusion without removing the catheter, aspirate residual drug through it, and administer the local antidote, with phentolamine the agent for which the most evidence exists, ideally within the first several hours, and topical nitroglycerin as an adjunct or temporizing option [29,30,31]. Across the larger observational series in which these precautions were applied, clinically significant extravasation was uncommon, and tissue necrosis was rare [26,31]. Translating this into routine practice requires defined nursing competencies, an antidote kit stocked and visible in the resuscitation area, and a clearly displayed extravasation algorithm. Importantly, there needs to be an event reporting system that feeds back into protocol refinement. These requirements are modest, but they are practical prerequisites for safe peripheral norepinephrine. They are most consequential in smaller hospitals and resource-limited settings, where peripheral administration may be the only timely option and where standardized competencies and a stocked antidote kit cannot be assumed.

#### 2.2.3. Barrier 3: The Recognition Problem Upstream of the Pressor Decision

The third barrier is the largest and the least often targeted. Time-to-vasopressor is a composite interval. In emergency department cohorts the time from arrival to effective hemodynamic support typically runs to one or two hours [8,9]. A multistate analysis of that interval localized the crowding-related delay to the initial assessment phase rather than the steps immediately preceding treatment: in a four-hospital study, crowding prolonged triage, clinician evaluation, and diagnostic data collection, while the interval between completed assessment and antibiotic initiation was essentially unaffected [32]. Independently, across 24 emergency departments, delay or non-completion of the core recognition steps (prompt triage, physician assessment, and continuous monitoring) predicted treatment delays exceeding 2.5 h [33]. The rate-limiting step is diagnostic, not therapeutic.

This changes how the supporting evidence should be read. If earlier norepinephrine tracks with survival in observational data, much of what is being captured is that earlier recognition tracks with survival, the same signal that produces the mortality gradient with time-to-treatment in mandated emergency sepsis care [34]. Recognition speed and execution speed cannot be separated within a single summary interval. They respond to different interventions: protocolized MAP thresholds and pre-authorized peripheral pressor orders compress the post-recognition interval, whereas triage screening criteria, reflex lactate sampling, and surveillance alerts wired into nursing workflow compress recognition itself.

The implication for the framework below is that both segments must be measured and addressed together. A program aimed only at the pressor decision will underperform, because most elapsed time accrues before recognition. A program aimed only at recognition will underperform if the downstream pathway stays discretionary and slow. A credible implementation design tracks recognition latency and execution latency separately rather than reporting one interval that obscures which half is failing.

Because recognition is the rate-limiting step, the upstream problem is also where emerging informatics tools may have the greatest effect. Automated electronic health record alerts, machine-learning classifiers, and continuous-monitoring early-warning systems are increasingly proposed to shorten time-to-recognition by surfacing at-risk patients before a clinician has formally identified sepsis. The evidence is mixed and should be read with care. In a prospective, multi-site evaluation, a machine-learning early-warning system was associated with reduced in-hospital mortality among septic patients whose alert was confirmed by a provider within three hours. This finding suggests real benefit when alerts are acted on promptly and embedded in workflow [35]. Of note, widely deployed proprietary tools such as the Epic Sepsis Model have shown poor external validation, limited sensitivity, and high false-alert rates in independent assessment, with alarm fatigue and clinician mistrust as recurrent failure modes [36]. An alert changes outcomes only when it is wired into a defined response pathway with clear ownership, rather than added as one more passive notification. Before these tools can be expected to close the recognition gap rather than widen the noise, they require prospective multicenter validation, attention to alert burden, and integration with the parallel resuscitation pathway proposed below.

### 2.3. A Parallel Resuscitation Framework with Protocolized Triggers

**Figure 1:**
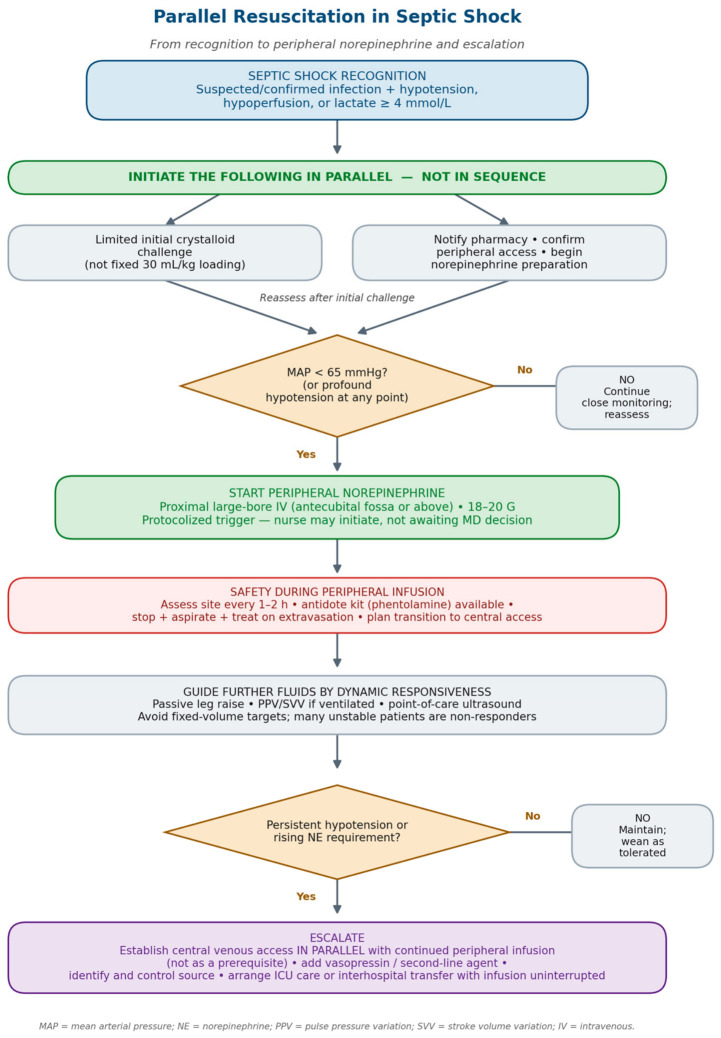
Parallel resuscitation algorithm for early septic shock, with protocolized triggers for concurrent fluid administration and peripheral norepinephrine initiation.

The current evidence base supports a resuscitation approach in which fluid administration and vasopressor preparation occur in parallel rather than in sequence. The operational version of this principle is summarized as an algorithm in Figure 1. One distinction should be made explicit before the framework that follows: the physiologic rationale and the timing evidence are established, whereas the parallel-resuscitation framework and the setting-stratified recommendations below are implementation-oriented proposals, that is, reasoned extrapolations intended to be tested rather than themselves trial-validated.

On septic shock recognition (suspected or confirmed infection with hypotension, hypoperfusion, or lactate ≥ 4 mmol/L), a limited initial crystalloid challenge, rather than fixed high-volume loading, is given. Simultaneously, pharmacy is notified, peripheral access is confirmed, and norepinephrine infusion preparation begins. This simultaneity is the core operational shift: when vasopressor initiation waits on central access, the additional time required for line placement can meaningfully delay treatment [10].

If MAP remains below 65 mmHg after the initial challenge, or immediately if hypotension is profound, peripheral norepinephrine is initiated through a proximal large-bore intravenous catheter sited in the antecubital fossa or above. The trigger is protocolized rather than discretionary. In a high-cognitive-load environment, discretionary triggers default to the most familiar pattern [37], which is fluids-first. A protocolized trigger also distributes the decision. The bedside nurse, recognizing that the threshold has been crossed, can prompt or initiate the next step rather than waiting for a physician decision in a crowded department.

Subsequent fluid administration is guided by dynamic indices of preload responsiveness rather than fixed-volume targets. These include passive leg raise, pulse pressure or stroke volume variation in mechanically ventilated patients, and point-of-care ultrasound. Fewer than half of hemodynamically unstable patients are fluid responsive, and continued fluid loading in non-responders is associated with worse outcomes [38]. Central venous access, when indicated, is established in parallel with continued peripheral pressor infusion rather than as a prerequisite. This framework does not advocate universal fluid restriction. It advocates individualized resuscitation in which fluid volume is titrated to physiology. Some patients, particularly those who are fluid-responsive or hypovolemic, will appropriately receive substantial volume, while others will not. The objection is to a fixed volume applied regardless of phenotype, not to fluids themselves.

None of this requires new evidence. It requires protocols that operate by default rather than by negotiation. The critical conceptual move is from discretionary to protocolized care. Protocolized resuscitation is not new to sepsis: early goal-directed therapy established the value of structured, time-anchored intervention [39], and although subsequent multicenter trials did not confirm benefit for its specific monitoring targets [40,41], they reaffirmed that early, organized treatment rather than any single hemodynamic endpoint is what drives outcome. The fluids-first sequence persists not because individual clinicians defend it but because, in a busy ED, the default pattern is the path of least cognitive resistance. Protocolized parallel resuscitation makes the alternative the default, which is the only intervention that reliably changes high-frequency clinical behavior at scale.

### 2.4. Stratified Implementation by Care Setting

The framework above is generic. Implementation differs across settings, and a review that ignored those differences would fail the clinicians it is intended to help. Table 1 summarizes the dominant barriers and highest-yield interventions by setting; the discussion below adds operational texture.

#### 2.4.1. U.S. Academic Centers

Academic centers typically have the infrastructure for early implementation: 24 h pharmacy, standing peripheral norepinephrine protocols, point-of-care ultrasound availability, and rapid-response or critical care consult coverage [42]. The dominant barrier is regulatory, namely SEP-1 compliance pressure and the institutional risk-aversion that flows from it. The most consequential interventions are institutional documentation templates that allow the SEP-1 fluid exception to be invoked without ad hoc justification, protocolized peripheral norepinephrine initiation with explicit MAP triggers, and integration of the parallel pathway into the existing sepsis order set as the default rather than as a deviation [43,44].

The sepsis order set is the specific operational lever. Many academic EDs operate sepsis pathways that, by structure, sequence fluids and vasopressors rather than parallelizing them: 30 mL/kg crystalloid, reassessment, pressor consideration. Reordering the same elements within an integrated parallel pathway requires no new technology and minimal additional training. It does require institutional consensus that the parallel pathway is the new default and that the SEP-1 exception will be invoked routinely when clinically appropriate. That consensus is best established by quality leadership in advance rather than negotiated by individual clinicians case by case.

#### 2.4.2. U.S. Community Emergency Departments

Community EDs face a different barrier mix. SEP-1 pressure is comparable, but the institutional resources for parallel resuscitation are often more limited. Pharmacy coverage may be intermittent, peripheral pressor protocols may not exist, and ICU transfer logistics may delay definitive care by hours [45]. Three interventions matter most: pre-specified protocols that do not require real-time pharmacist or critical care input, standing orders for peripheral norepinephrine on defined MAP triggers, and clear transfer pathways that allow pressor infusion to continue uninterrupted across the transfer.

The peripheral-vasopressor culture barrier is more pronounced in this setting than at academic centers [42]. Closing it requires explicit institutional endorsement, repeated in-service education, visible nursing leadership, and clinical exemplars who normalize the practice. Policy alone is insufficient. Transfer logistics deserve specific attention. The interval between septic shock recognition in a community ED and arrival at the receiving ICU can stretch to several hours, and that interval is precisely the window in which early peripheral norepinephrine, initiated and continued through transport, may alter the trajectory most. System-level coordination, pre-existing transfer agreements, paramedic comfort with continuous norepinephrine infusion, and receiving-facility willingness to accept patients on peripheral pressors are what convert an inherently delayed pathway into a continuous one [45].

#### 2.4.3. Resource-Limited International Settings

International settings vary widely in resource availability. In settings with limited ultrasound, intermittent laboratory access, and high ED volumes, simpler protocolized triggers are more practical than individualized hemodynamic assessment. The argument for early peripheral norepinephrine is in some ways strongest here, because the alternative, waiting for central access, advanced monitoring, or ICU availability, implies delays measured in hours rather than minutes. A simplified framework requiring only blood pressure measurement, basic clinical assessment, peripheral intravenous access, and norepinephrine availability is plausibly feasible in most settings. It may offer a large absolute benefit, though this expectation requires local validation rather than assumption. International protocol development should privilege simplicity over physiologic precision.

A frequently underappreciated point is that international critical care literature sometimes treats the implementation gap as a problem of resource scarcity that high-income settings have already solved. The opposite is closer to the truth. High-income settings have access to every component of an early-vasopressor pathway and frequently still fail to deliver it expeditiously [9,10]. The implementation problem is principally one of protocol design, regulatory alignment, and institutional culture. Settings with fewer resources may, paradoxically, find it easier to install a simple parallel pathway because they carry fewer of the structural constraints that complicate U.S. practice.

International experience also offers useful comparators. Other health systems have confronted the same recognition-and-delivery gap with different regulatory instruments. In England, sepsis improvement has been pursued largely through financial incentives for screening and timely treatment, such as the Commissioning for Quality and Innovation indicators introduced in 2015, alongside national early-warning scoring. This approach raised the profile of screening but, like SEP-1, anchored measurement to antibiotics and bundle completion rather than to early hemodynamic support. Reliable measurement has been hampered by the absence of a dedicated way to capture sepsis in routine data, prompting reliance on administrative “suspicion of sepsis” surrogates instead [46]. Quality-improvement programs elsewhere illustrate what changes behavior when a measure is paired with workflow redesign. A multicenter program across hospitals in the Lombardy region of Italy improved adherence to a simplified one-hour bundle and was independently associated with lower in-hospital mortality, with the gains concentrated in the subgroups where bundle adherence actually rose [47]. In Denmark, a structured early-warning trigger system embedded in routine vital-sign monitoring was associated with a higher proportion of septic patients receiving appropriate antibiotics before ICU admission. This is an instructive parallel to the upstream recognition problem discussed above [48]. Two themes recur across these settings. First, the specific barrier differs by system (financial incentives in England, regional quality programs in Italy, surveillance-based recognition in Denmark), with the underlying lesson being consistent—a measure or alert changes outcomes only when it is wired into a defined response pathway. Second, none of these programs has made early vasopressor initiation a tracked endpoint alongside fluids and antibiotics. The implementation gaps this review describes is therefore not unique to the United States; it recurs, in system-specific form, wherever sepsis policy treats restoration of vascular tone as a later step rather than a concurrent priority.

### 2.5. What This Framework Does Not Resolve

Several questions remain genuinely uncertain. The optimal MAP target in the first hour is not settled; SEPSISPAM supports a default of 65 mmHg [49], but individual patient targets vary, and the 2026 Surviving Sepsis Campaign guidelines now suggest a 60–65 mmHg range specifically for adults aged 65 and older [19]. The optimal timing of vasopressin or other second-line agents in patients with rising norepinephrine requirements remains debated [19]. Whether dynamic preload-responsiveness assessment should formally gate fluid administration in the ED, rather than guide it, has not been tested in randomized fashion [38].

These uncertainties are about how to optimize the approach, not whether to take it. The first-order question, whether earlier vasopressor initiation is generally better than later, has been answered consistently across multiple study designs and settings. Acting on that answer does not require resolving the optimization questions first, just as the implementation of early antibiotics in sepsis did not wait for resolution of the optimal antibiotic choice [34,50].

## 3. Conclusions

The case for early vasopressor initiation in septic shock no longer rests on a single trial or contested physiologic model. It rests on convergent randomized, observational, and physiologic evidence accumulated over more than a decade. The persistent gap between that evidence and bedside practice is now the field’s central problem, and it is not closed by adding another physiologic argument to the literature. It is closed by addressing the regulatory incentives that codify the fluids-first sequence, the institutional and cultural friction that delays peripheral pressor initiation, and the upstream recognition problem for which pressor timing is partly a surrogate.

Three priorities follow. First, U.S. sepsis quality measures should be re-examined to determine whether early vasopressor initiation can achieve the perfusion-restoration aim of the fluid bundle in patients for whom large-volume crystalloid loading is physiologically inappropriate [16]. Second, institutional protocols and nursing workflows should be redesigned to make protocolized parallel resuscitation the default. Third, international protocol development should privilege simplicity, validate locally, and avoid the structural baggage of adapted high-resource pathways.

What remains unsettled is whether the systems in which septic shock is recognized and treated will be reorganized to act on this evidence. Addressing that organizational gap, rather than generating further physiologic data, is the field’s most consequential next step.

## Figures and Tables

**Table 1:** Setting-stratified implementation of early vasopressor therapy in septic shock.

Highest-Yield Interventions	Dominant Barrier	Care Setting
Documentation templates for the SEP-1 fluid exception; protocolized peripheral norepinephrine with explicit MAP triggers; parallel pathway as the default in the sepsis order set	Regulatory: SEP-1 compliance pressure and institutional risk-aversion despite available infrastructure	U.S. academic centers
Standing orders for peripheral norepinephrine on defined MAP triggers; nursing-initiated lactate measurement at triage; transfer pathways that preserve continuous infusion; in-service education and visible nursing leadership	Mixed: SEP-1 pressure plus limited pharmacy and ICU resources; pronounced peripheral-pressor culture barrier	U.S. community emergency departments
Simplified protocols requiring only blood pressure, clinical assessment, peripheral access, and norepinephrine; local validation rather than adapted high-resource pathways	Resource scarcity (ultrasound, lab access, ICU capacity) but fewer structural constraints than U.S. settings	Resource-limited international settings

MAP = mean arterial pressure; SEP-1 = Centers for Medicare and Medicaid Services Severe Sepsis and Septic Shock Management Bundle.

## Data Availability

No new data were created or analyzed in this study.
